# Association between Cervical Spondylosis and Migraine: A Nationwide Retrospective Cohort Study

**DOI:** 10.3390/ijerph15040587

**Published:** 2018-03-25

**Authors:** Wang-Sheng Lin, Tung-Fu Huang, Tien-Yow Chuang, Cheng-Li Lin, Chia-Hung Kao

**Affiliations:** 1 Department of Physical Medicine and Rehabilitation, Taipei Veterans General Hospital, Taipei 11217, Taiwan; a8889bms47@gmail.com (W.-S.L.); tychuang@vghtpe.gov.tw (T.-Y.C.); 2 Department of Surgery, Faculty of Medicine, National Yang-Ming University, Taipei 11221, Taiwan; tungfuh@yahoo.com.tw; 3 Department of Orthopaedics & Traumatology, Taipei Veterans General Hospital, Taipei 11217, Taiwan; 4 Management Office for Health Data, China Medical University Hospital, Taichung 404, Taiwan; orangechengli@gmail.com; 5 College of Medicine, China Medical University, Taichung 404, Taiwan; 6 Graduate Institute of Clinical Medical Science and School of Medicine, College of Medicine, China Medical University, No. 2, Yuh-Der Road, Taichung 404, Taiwan; 7 Department of Nuclear Medicine and PET Center, China Medical University Hospital, Taichung 404, Taiwan; 8 Department of Bioinformatics and Medical Engineering, Asia University, Taichung 413, Taiwan

**Keywords:** cervical spondylosis, migraine, retrospective cohort study, population-based

## Abstract

***Background:*** Few studies have investigated the longitudinal association between cervical spondylosis (CS) and migraine by using a nationwide population-based database. ***Methods:*** We conducted a retrospective cohort study from 2000 to 2011 identifying 27,930 cases of cervical spondylosis and 111,720 control subjects (those without cervical spondylosis) from a single database. The subjects were frequency-matched on the basis of sex, age, and diagnosis date. The non- cervical spondylosis cohort was four times the size of the cervical spondylosis cohort. To quantify the effects of cervical spondylosis on the risk of migraine, univariate and multivariate Cox proportional hazard regression analyses were used to calculate the hazard ratio (HR), and 95% confidence interval (CI). ***Results:*** After a 10-year follow-up controlling for potential confounding factors, overall migraine incidence was higher in the cervical spondylosis cohort than in the non-cervical spondylosis cohort (5.16 and 2.09 per 1000 people per year, respectively; crude hazard ratio = 2.48, 95% confidence interval = 2.28–2.69), with an adjusted hazard ratio of 2.03 (95% confidence interval = 1.86–2.22) after accounting for sex, age, comorbidities, and medication. Individuals with myelopathy in the cervical spondylosis cohort had a 2.19 times (95% confidence interval = 1.80–2.66) higher incidence of migraine when compared than did those in the non- cervical spondylosis cohort. ***Conclusions:*** Individuals with cervical spondylosis exhibited a higher risk of migraine than those without cervical spondylosis. The migraine incidence rate was even higher among individuals with cervical spondylotic myelopathy.

## 1. Introduction

Migraine is the most prevalent and incapacitating neurovascular disorder worldwide, affecting approximately one billion people, exerting a considerable impact on quality of life, and representing a significant socioeconomic burden [1,2,3]. Speculated origins of pain are cortical neuronal hyperexcitability [4], modulatory dysfunction of brainstem and diencephalic systems [5], and peripheral activation [6], all of which lead to the release of vasoactive neuropeptides in the trigeminovascular system to process pain [7,8].

Although many hypotheses regarding migraine triggers have been proposed, the significance of causal relationships between the triggers is obscured [9,10]. Among these triggers, cervical pathologies may initiate the sequence of events that results in migraine symptoms. The extensive functional convergence of upper cervical spinal cord from the descending fibers in the trigeminal nucleus caudalis, which terminates within the trigeminocervical nucleus, and the afferent fibers from the upper cervical roots, which communicate in this region, accounting for the bi-directional pathway of pain between the neck and head. This interaction refers the cervical pathologies to the head, which is the activity also proposed to cause cervicogenic headache [11]. Constantly noxious cervical afferent irritation via this pathway is a possible key element in causing migraines. 

Neck pain and muscle tension are common migraine symptoms and both could be sequelae of neck injuries, according to the musculoskeletal anatomy [11,12]. Moreover, administering multiple injections in targeted head and neck regions is sometimes considered important for the management of migraines, cervicogenic headaches, and myofascial referred pain [11,13,14,15,16], indicating that headache and neck pain may share some common pathways. 

Although a previous report indicated that cervical spondylosis (CS) accounts for 15.9% of migraineurs [17], until now epidemiological evidence of a link between CS and the risk of migraine is minimal. Therefore, we conducted this nationwide retrospective cohort study to investigate the longitudinal causal relationship between CS and migraines and CS severity in relation to the risk of developing a migraine.

## 2. Methods

### 2.1. Data Source

The data used in this retrospective study was retrieved from the Longitudinal Health Insurance Database 2000 (LHID2000) of Taiwan, a subdataset of the National Health Insurance Research Database (NHIRD) that comprises 1,000,000 randomly selected people from the NHIRD. The NHIRD of Taiwan contains the detailed health care data of more than 99% of the Taiwanese population (more than 23.74 million residents) [18]. The LHID2000 has been successfully applied in numerous studies [19,20]. The diagnostic coding in our study was according to the Ninth Revision ICD-9-CM (the International Classification of Diseases-Clinical Modification). Our study was approved by the IRB (Institutional Review Board) in China Medical University (CMUH104-REC2-115-CR2). Because the patients’ personal information is anonymous without identification numbers, therefore the informed consent was not required. The dataset is belonging to the Taiwan Ministry of Health and Welfare (MOHW) 

### 2.2. Sampled Participants

We selected individuals aged ≥18 years diagnosed with CS without (ICD-9-CM code 721.0) or with (ICD-9-CM code 721.1) myelopathy from 2000 to 2010 as the CS cohort. The diagnosis time of the CS cohort was defined to be the index date. We randomly selected the non-CS cohort (the control subjects) at a 4:1 ratio from the database to match with the CS cohort. Participants in both of the cohorts with previous migraine diagnoses (ICD-9-CM code 346) were excluded. The medical fraud by overbilling for health care or cheating charges based on incorrect diagnoses were avoided carefully, and the NHI Bureau in Taiwan monitored the disease coding strictly. As aforementioned, highly prevention from the coding errors, misdiagnosis, or improper therapy supported the reliability of NHIRD data to explore the risk of migraine in CS patients. Moreover, the diagnosis of migraine based on the International Classification of Headache Disorders (ICHD-3) criteria (the 3rd edition) was only made by the professional neurologists in Taiwan, which increased the confidence of the accuracy [21]. Regarding the diagnosis of CS with myelopathy, it required a careful correlation between findings from the nature history and clinical examination, supported by radiological evidence showing spinal cord compression. Furthermore, the NHI regulated the coding and audited insurance reimbursement claims routinely. 

### 2.3. Outcome and Comorbidities

We followed the participants in the CS and non-CS cohorts until migraine diagnoses were made or the participants were censored because of withdrawal from the National Health Insurance (NHI) program or until 31 December 2011. Pre-existing comorbidities for each individual included hypertension (ICD-9-CM codes 401–405), hyperlipidemia (ICD-9-CM code 272), depression (ICD-9-CM codes 296.2–296.3, 300.4, 311), coronary artery disease (ICD-9-CM codes 410–414), anxiety (ICD-9-CM codes 300.0, 300.2, 300.3, 308.3, 309.81), sleep disorders (ICD-9-CM codes 307, 780.5), irritable bowel syndrome (ICD-9-CM code 564.1), diabetes (ICD-9-CM code 250), and fibromyalgia (ICD-9-CM code 729.1).

### 2.4. Statistical Analysis

We used Chi-square tests and Student’s *t* tests to compare sex, age, and comorbidities between the CS and non-CS cohorts. Cumulative incidences of migraines in both of the cohorts were compared using the Kaplan-Meier method and variations between the two cohorts were compared using a log-rank test. We calculated the incidence rate of migraines based on various risk factors and stratified the results based on sex, age, and comorbidities. We used Cox proportional hazard regression models to measure the hazard ratios (HRs) and 95% confidence intervals (CIs) to assess the risk of migraine after adjustments for age, sex, and the related comorbidities. In addition, the relative risk of migraine development stratified based on sex, age, and comorbidities in both of the cohorts was analyzed for comparison. These above statistical measurements were analyzed based on SAS (version 9.4 for Windows, SAS Institute, Cary, NC, USA).

## 3. Results

**Figure 1:**
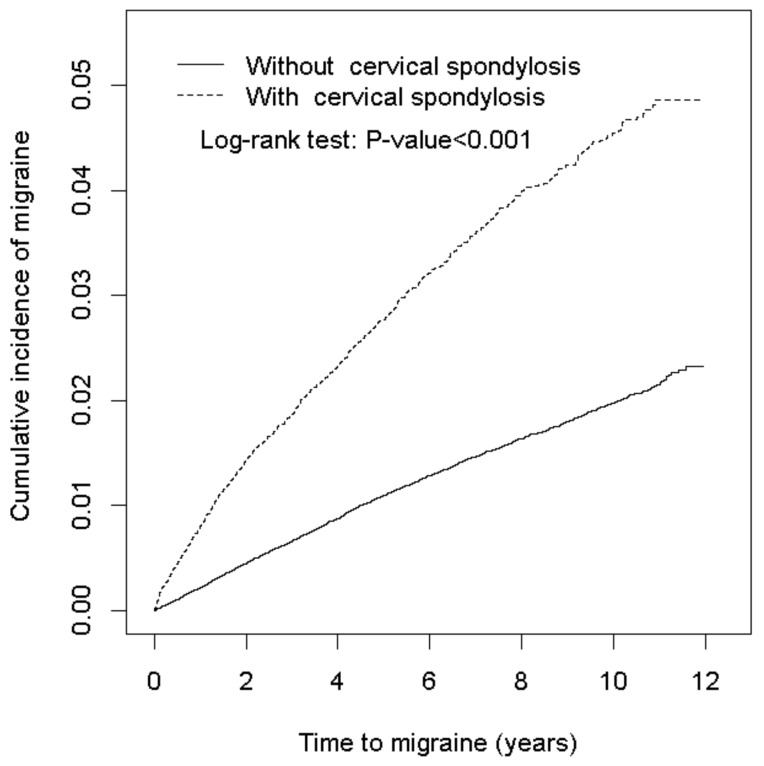
Cummulative incidence of migraine in individuals with and without cervical spondylosis (CS).

The CS and non-CS cohorts comprised 27,930 and 111,720 cases, respectively. Among all of the participants, 56.9% were women and 73.7% were aged under 64 years (Table 1). The mean ages of both cohorts were 44.8 ± 16.6 (CS) and 44.5 ± 16.8 years (non-CS). The CS cohort exhibited higher prevalence of all baseline comorbidities. During the mean follow-up of 6.13 (standard deviation (SD) = 3.19) years for the CS cohort and 6.07 (SD = 3.20) years for the non-CS cohort, the cumulative incidence of migraines was higher among individuals with CS than those without (log-rank test *p* < 0.001) (Figure 1).

The migraine incidence rates were 5.16 and 2.09 per 1000 people per year in the CS and non-CS cohorts, respectively (Table 2). The risk of migraine was higher in the CS cohort than in the non-CS cohort (adjusted HR (aHR) = 2.03, 95% CI = 1.86–2.22). When compared with individuals that are aged ≥65 years, individuals aged 20–49 years (aHR = 1.81, 95% CI = 1.58–2.06) and 50–64 years (aHR = 1.40, 95% CI = 1.24–1.58) had a higher risk of developing migraine. The aHR values revealed that the risk of developing migraine was 1.92 times higher among women than men. The multivariate Cox model revealed that the risk of migraine was higher among individuals with the comorbidities of hypertension, hyperlipidemia, depression, coronary artery disease, anxiety, sleep disorders, irritable bowel syndrome, diabetes, and fibromyalgia.

The incidence and risk of migraine in both cohorts were compared based on the variables of sex, age, and comorbidities (Table 3). For all of the variables, the risk of migraine remained higher in the CS cohort than those in the non-CS cohort.

Table 4 shows the incidence and aHR values of migraine associated with different various forms of CS. The participants with myelopathy in the CS cohort exhibited a 2.19 times higher risk of migraine (95% CI = 1.80–2.66) than those in the non-CS cohort, whereas those without myelopathy exhibited a 2.01 times higher risk in the CS cohort (95% CI = 1.83–2.20). As noted, the age intervals used to stratify were under 49 years, from 50 to 64 years, above 65 years. The numbers for each age cohort are 1293(35.5%), 1302(35.7%), and 1048(28.8%), respectively.

## 4. Discussion

This study is the first to use a population-based database to demonstrate the long-term risk of migraine in individuals with CS. The primary findings support our hypothesis that individuals with CS are at a higher risk of developing migraine than those without CS. The cumulative migraine frequency at the conclusion of the follow-up period was higher in the CS cohort than in the non-CS group. The migraine rates among 1000 people per year were 5.16 and 2.09 in the CS and non-CS cohorts, respectively. Moreover, the CS cohort exhibited a 2.03 times higher risk of migraine (95% CI = 1.86–2.22) than the non-CS cohort. Furthermore, in the CS cohort, the participants with myelopathy were at a higher risk of developing a migraine than those without myelopathy.

Cervical musculoskeletal abnormalities and headache disorders were a bi-directional comorbidity, with nearly two-thirds of migraineurs coexisting with neck pain or stiffness, and one-fifth of patients with cervicogenic headache in pain clinics [22,23]. Previous studies have disclosed that almost 90% patients with headache accepted anterior cervical operation for the treatment of symptoms with cervical myelopathy or radiculopathy [24,25]. Furthermore, the authors observed that patients with headache disorders presented the myofascial trigger points, clear-cut areas of muscle tenderness, and posture changes, when compared with non-headache control [22,26]. Thus, our results corroborate those of previous studies on the linking between cervical spine disorder and migraine, emphasizing the importance of assessment for migraine at first and follow-up period in CS patients due to the increasing risk for the development of migraine.

Regarding the effects of age and gender on migraine development, our results correlate with those of previous studies [3,27], which have demonstrated that young and middle-aged women with CS develop migraine more easily than do other individuals. Adjustments for the factors of sex, age, hypertension, hyperlipidemia, depression, anxiety, sleep disorders, coronary artery disease, irritable bowel syndrome, and fibromyalgia achieved statistical significance. Only the result of diabetes was insignificant. Although CS is an age-related disorder affecting the discs and vertebrae of the cervical spine, notably, the HR of the migraine occurrence did not increase with age [28]. First, we assumed that the inflammatory effects of the nucleus rather than the degenerative factor in female adults that are aged under 50 years might play a role in migraine development [28]. Persistent peripheral nociceptive impulses may induce neuroplastic changes in the spinal cord and brain, causing central sensitization and pain. Therefore, the combination of tonic nociceptive input and central disinhibition may play a role in migraine development [29]. In vivo, a slow progressive cord irritation resulted in complement-mediated response, contributing to the synapse destruction, neuronal and oligodendrocyte death [30,31]. Moreover, recent imaging advances also demonstrated that patients with more severe CS correlated with higher inflammation volumes [32]. The findings indicated that inflammatory cascade might serve as the potential molecular mechanisms underlying the pathogenesis of cervical spondylotic myelopathy, leading to migraine attack. Second, increased inflammation in the cervical spine due to the proinflammatory molecules release into the bloodstream upregulated the hypothalamic-pituitary-adrenal axis, causing the hypothalamic dysfunction [33]. This process intensified the sensitivity to pain in the brain, which may lead to predisposition to migraine.

Understanding of the precise mechanisms of the relationship between CS and migraine risk remains limited. Cervical vertebral degenerative processes can compromise the capsular ligaments of facet joints, thereby contributing to the hypermobility of upper cervical vertebrae [34]. Such cervical instability causes the dysregulation of the vertebrobasilar arteries, which leads to migraines [35,36]. Watson and Drummond assessed the effects of sustained pressure on the atlanto-occipital segments and C2-3 zygapophyseal joints to relieve migraines [37]. In addition, several studies have reported that myofascial trigger points could reproduce migraine symptoms [38,39], Moreover, an increasing amount of evidence shows that dyscoordination between the dorsal horns of the upper cervical spinal cord and trigeminal nucleus caudalis may induce cervicogenic headaches. The prolonged irritated inputs implicate in the pain processing through trigeminovascular system, which can provoke and worsen the symptoms of migraine [40,41].

Regarding CS severity, our results revealed in the CS cohort, the participants with myelopathy may be at a greater risk of developing migraine than those without myelopathy. Revanappa et al. demonstrated that more than 50% of individuals with cervical spondylotic myelopathy also had concomitant autonomic dysfunction [42], where sympathetic postganglionic fibers exit through cervical posterior longitudinal ligaments [43]. In these individuals, once the ligaments had been compressed, abnormal activities in the middle cervical ganglia occurred [44], which could partially explain the progression of autonomic dysfunction due to cervical spondylotic myelopathy. Such structural and functional alternations in the cervical spinal cord may upregulate the associated nociceptive expressions, which could contribute to migraine development. 

Our study has several clinical implications in public health. Firstly, clinicians should assess CS patients for migraine from beginning to follow-up because of the risk for developing the disorder. Secondly, as cervical spondylosis increased the risk of migraine, particularly in young to middle-aged women with CS and comorbidities, physicians should do the headache survey carefully for those susceptible patients. Thirdly, our results demonstrated that migraine incidence rate was even higher among individuals with cervical spondylotic myelopathy, which suggested that additional research is needed to evaluate the influence of CS severity.

Although this study has the advantage of feasibility and generalizability because of the large sample size and low dropout rate, it had several flaws. Firstly, the NHIRD has limitations, particularly in relation to biographical information, such as clinical features, imaging findings, laboratory studies, personal lifestyle, and medication, all of which are irretrievable. Secondly, the diagnostic accuracy of CS and migraine were totally dependent based on ICD-9-CM codes. In theory, the diagnosis of CS with or without myelopathy should be on the basis of the appropriate imaging evidence together with physical examinations and history taking. Generally, neurologists in Taiwan developed a headache diary as well as a structured intake form to do the headache survey (clinical headache pattern, psychological comorbidities, medication use and treatment response, functional disability, and quality of life) [45], the diagnosis of migraine based on the International Classification of Headache Disorders (ICHD-3) criteria (the 3rd edition). However, no one could confirm whether patients with the coding were accurately diagnosed due to the inherent weakness for all of the studies based on databank. Nonetheless, our results remain reliable because the NHI administration ministry randomly samples the medical charts routinely, verifying the coding in NHIRD dataset from every contracted medical institution. Thirdly, our database was derived from the NHI program in Taiwan, which predominantly enrolls Taiwanese citizens. The genetic factor of our participants would have been minimized in our study. However, there was no corresponding ICD-9-CM code for any particular type of migraine. Relationship between CS and any particular type of migraine (with and without aura) could not be substantiated, the more definite coding is required to strengthen the association between CS and migraine subtype.

## 5. Conclusions

CS increases the risk of migraine, especially in individuals with cervical spondylotic myelopathy. We showed that young to middle-aged women with CS and comorbidities were at the highest risk of migraine. Although the precise underlying mechanisms of this longitudinal association remain unknown, our results indicated that these two entities are closely related. However, in daily clinical practice, this truth will not come to light, multimodal management of migraine is highly required by the clinical practitioners.

## Figures and Tables

**Table 1:** Comparisons of demographic characteristics and comorbidities in individuals with and without CS.

Variable	Cervical Spondylosis		
	No	Yes	Standard Mean Difference
	(*N* =111,720)	(*N* =27,930)	
**Sex**			0.99
Women	63,592(56.9)	15,898(56.9)	
Men	48,128(43.1)	12,032(43.1)	
**Age stratified**			0.99
≤49	40,452(36.2)	10,113(36.2)	
50–64	41,880(37.5)	10,470(37.5)	
65+	29,388(26.3)	7347(26.3)	
**Age, mean ± SD ^a^**	55.1(14.0)	55.6(13.6)	<0.001
**Comorbidity**			
Hypertension	36,886(33.0)	11,658(41.7)	<0.001
Hyperlipidemia	22,730(20.4)	8887(31.8)	<0.001
Depression	4498(4.03)	2252(8.06)	<0.001
Coronary artery disease	17,160(15.4)	6941(24.9)	<0.001
Anxiety	10,300(9.22)	5607(20.1)	<0.001
Sleep disorder	20,851(18.7)	9450(33.8)	<0.001
Irritable bowel syndrome	4969(4.45)	2487(8.90)	<0.001
Diabetes	10,876(9.74)	3182(11.4)	<0.001
Fibromyalgia	4599(4.12)	2984(10.7)	<0.001

Chi-square test; ^a^ *t* test; CS cohort follow-up time mean = 6.13 (3.19); Non-CS cohort follow-up time mean = 6.07 (3.20).

**Table 2:** Migraine incidences and risk factors.

Variable	Event	PY	Rate ^#^	Crude HR (95% CI)	Adjusted HR ^&^ (95% CI)
**Cervical spondylosis**					
No	1414	677,913	2.09	1.00	1.00
Yes	883	171,179	5.16	2.48(2.28, 2.69) ***	2.03(1.86, 2.22) ***
**Age group, year**					
20−49	997	330,050	3.02	1.46(1.31, 1.64) ***	1.81(1.58, 2.06) ***
50−64	874	317,220	2.76	1.32(1.18, 1.48) ***	1.40(1.24, 1.58) ***
≥65	426	201,822	2.11	1.00	1.00
**Sex**					
Female	1719	494,959	3.47	2.14(1.95, 2.35) ***	1.92(1.74, 2.11) ***
Male	578	354,133	1.63	1.00	1.00
**Comorbidity**					
**Hypertension**					
No	1465	570,275	2.57	1.00	1.00
Yes	832	278,816	2.98	1.15(1.06, 1.25) **	1.06(0.95, 1.18)
**Hyperlipidemia**					
No	1698	668,229	2.54	1.00	1.00
Yes	599	180,862	3.31	1.29(1.17, 1.41) ***	1.01(0.91, 1.12)
**Depression**					
No	2090	813,829	2.57	1.00	1.00
Yes	207	35,263	5.87	2.24(1.94, 2.58) ***	1.13(0.97, 1.32)
**Coronary artery disease**					
No	1800	711,569	2.53	1.00	1.00
Yes	497	137,523	3.61	1.42(1.28, 1.56) ***	1.20(1.07, 1.35) **
**Anxiety**					
No	1785	764,303	2.34	1.00	1.00
Yes	512	84,789	6.04	2.53(2.30, 2.80) ***	1.48(1.32, 1.66) ***
**Sleep disorder**					
No	1444	690,750	2.09	1.00	1.00
Yes	853	158,342	5.39	2.52(2.31, 2.74) ***	1.81(1.64, 1.99) ***
**Irritable bowel syndrome**					
No	2105	809,522	2.60	1.00	1.00
Yes	192	39,569	4.85	1.83(1.57, 2.12) ***	1.24(1.06,1.44) **
**Diabetes**					
No	2112	775,714	2.72	1.00	1.00
Yes	185	73,378	2.52	0.91(0.78, 1.05)	-
**Fibromyalgia**					
No	2127	808,699	2.63	1.00	1.00
Yes	170	4093	4.21	1.57(1.34, 1.83) ***	1.08(0.92, 1.27)

CI: confidence interval; HR: hazard ratio; PY: people per year; ^#^: incidence rate per 1000 people per year; ^&^: model was adjusted for age, sex, and the comorbidities of hypertension, hyperlipidemia, depression, coronary artery disease, anxiety, sleep disorders, irritable bowel syndrome, and fibromyalgia by using Cox proportional hazards regression; **p < 0.01; ***p < 0.001.

**Table 3:** Comparison of incidence rate of migraine hazard ratios (HR) between individuals with and without CS based on demographic characteristics and comorbidities.

Variable	Cervical spondylosis							
	No			Yes			Crude HR (95% CI)	Adjusted HR ^&^ (95% CI)
	Event	PY	Rate ^#^	Event	PY	Rate ^#^		
**Sex**								
Women	1082	395,676	2.73	637	99,282	6.42	2.35(2.13, 2.59) ***	1.93(1.74, 2.14) ***
Men	332	282,237	1.18	246	71,896	3.42	2.91(2.47, 3.44) ***	2.32(1.95, 2.76) ***
**Stratify age**								
≤49	620	264,087	2.35	377	65,963	5.72	2.44(2.14, 2.77) ***	1.94(1.70, 2.23) ***
50–64	532	253,619	2.10	342	63,600	5.38	2.56(2.24, 2.94) ***	2.05(1.78, 2.36) ***
65+	377	160,207	1.64	164	41,615	3.94	2.42(1.99, 2.94) ***	2.08(1.70, 2.55) ***
**Comorbidity ^‡^**								
No	520	342,246	1.52	180	49,454	3.64	2.40(2.02, 2.84) ***	2.36(1.99, 2.80) ***
Yes	894	335,667	2.66	703	121,725	5.78	2.19(1.98, 2.41) ***	2.10(1.91, 2.32) ***

Rate ^#^: incidence rate per 1000 people per year; Crude HR: relative hazard ratio; Adjusted HR ^&^: crude HR mutually adjusted for age, sex, and the comorbidities of hypertension, hyperlipidemia, depression, coronary artery disease, anxiety, sleep disorders, irritable bowel syndrome, and fibromyalgia by using Cox proportional hazards regression; Comorbidity ^‡^: individuals with any one of the comorbidities of hypertension, hyperlipidemia, depression, coronary artery disease, anxiety, sleep disorders, irritable bowel syndrome, diabetes, and fibromyalgia were classified in the comorbidity group; *** *p* < 0.001.

**Table 4:** Migraine incidences and HRs among individuals with various types of CS and those without CS.

Variable	N	Events	PYs	Rate ^#^	Crude HR (95% CI)	Adjusted HR ^&^ 95% CI)
Without cervical spondylosis	111,720	1414	677,913	2.09	1.00	1.00
Type of Cervical spondylosis						
Cervical spondylosis without myelopathy	24,287	771	150,298	5.13	2.47(2.26, 2.69 ) ***	2.01(1.83, 2.20) ***
Cervical spondylosis with myelopathy	3643	112	20,880	5.36	2.55(2.10, 3.09) ***	2.19(1.80, 2.66) ***

Rate ^#^: incidence rate per 1000 people per year; Crude HR: relative hazard ratio; Adjusted HR ^&^: crude HR mutually adjusted for age, sex, and the comorbidities of hypertension, hyperlipidemia, depression, coronary artery disease, anxiety, sleep disorders, irritable bowel syndrome, and fibromyalgia by using Cox proportional hazards regression; *** *p* < 0.001

## Abbreviations

CS	cervical spondylosis
HR	hazard ratio
CI	confidence interval
NHIRD	National Health Insurance Research Database
LHID2000	Longitudinal Health Insurance Database 2000
ICD-9-CM	International Classification of Diseases, Ninth Revision, Clinical Modification
